# Locomotor circumvention strategies are altered by stroke: II. Postural Coordination

**DOI:** 10.1186/s12984-017-0265-7

**Published:** 2017-06-15

**Authors:** Anuja Darekar, Anouk Lamontagne, Joyce Fung

**Affiliations:** 1 School of Physical and Occupational Therapy, Faculty of Medicine, McGill University, Montreal, QC Canada; 2 Feil and Oberfeld Research Center, Jewish Rehabilitation Hospital of the Centre Intégré de Santé et Services Sociaux de Laval (CISSS-Laval); Research site of the Montreal Centre for Interdisciplinary Research in Rehabilitation (CRIR), 3205, Place Alton Goldbloom, Laval, QC H7V 1R2 Canada

**Keywords:** Collision avoidance, Gait, Cerebrovascular accident, Walking adaptation, Coordination

## Abstract

**Background:**

Locomotor strategies for obstacle circumvention require appropriate postural coordination that depends on sensorimotor integration within the central nervous system. It is not known how these strategies are affected by a stroke. The objective of this study was to contrast postural coordination strategies used for obstacle circumvention between post-stroke participants (*n* = 12) and healthy controls (*n* = 12).

**Methods:**

Participants walked towards a target in a virtual environment (11 × 8 m room) with cylindrical obstacles that were stationary or approaching from head-on, or diagonally 30° left/right.

**Results:**

Two stepping strategies for obstacle circumvention were identified: 1) side step: increase in step width by the foot ipsilateral to the side of circumvention; 2) cross step: decrease in step width by the foot contralateral to the side of circumvention. The side step strategy was favoured by post-stroke individuals in circumventing stationary and head-on approaching obstacles. In circumventing diagonally approaching obstacles, healthy controls generally veered opposite to obstacle approach (>60% trials), whereas the majority of post-stroke participants (7/12) veered to the same side of obstacle approach (V_same_). Post-stroke participants who veered to the opposite side (V_opp_, 5/12) were more independent and faster ambulators who favoured the side step strategy in circumventing obstacles approaching from the paretic side and cross step strategy for obstacles approaching from the non-paretic side. V_same_ participants generally favoured the side step strategy for both diagonal approaches. Segmental rotation amplitudes and latencies were largest in the V_same_ group, and significantly greater in post-stroke participants than controls for all obstacle conditions. All participants initiated circumvention with the feet followed by the pelvis and thorax, demonstrating a caudal-rostral sequence of reorientation.

**Conclusion:**

Postural coordination strategies for obstacle circumvention were altered post stroke, depending on the residual or restored functional abilities. Segmental re-orientations are also affected by the motion and direction of obstacle.

## Background

Collision-free navigation around stationary and dynamic obstacles is an important component of safe community ambulation. Although circumventing obstacles often requires changing walking direction, the segmental coordination involved is quite different as compared to turning towards a new goal. . While a change in walking direction towards a new goal is typically initiated with head and gaze orientation towards the intended walking direction, followed by the trunk or body’s center of mass (CoM), and lastly the feet [1, 2], obstacle circumvention involves trunk yaw that is either preceded or followed by head yaw and greater contribution from the foot segment in executing the transient directional change [3]. The difference in coordination strategies observed in the two tasks suggests that locomotor adaptations may be shaped by the constraints imposed by the task, environment as well as the individual. Different subject populations show coordination strategies that are dissimilar to those seen in healthy young adults. For instance, children use similar coordination strategies for obstacle circumvention and changing walking direction towards a new goal [4]; while older adults initiated segmental reorientation earlier as compared to young adults [5] to ensure safe circumvention.

Further, it is known that obstacle characteristics (stationary or mobile, direction of motion) may affect circumvention strategies in young adults [6, 7] and post-stroke individuals [8] such as clearance and preferred direction of circumvention. However, the extent of postural coordination required for safe circumvention post stroke is not known. Therefore, we aimed to compare postural coordination strategies required for circumventing obstacles under different conditions: (1) stationary vs. moving; (2) approaching diagonally from left vs. right.

## Methods

### Participants

Both post-stroke and healthy participants were recruited through flyers posted at various public locations in the hospital premises of the Jewish Rehabilitation Hospital, in Laval (greater Montreal), Canada. In addition, medical charts were screened for inclusion criteria mentioned below to identify potential participants with stroke. Healthy participants included individuals from the community or volunteers at the Jewish Rehabilitation Hospital. Potential participants were first contacted by secretarial staff not related to the study to give preliminary information about the study and to obtain verbal consent to be contacted by the researchers. After obtaining verbal consent, the first author contacted potential participants to provide detailed information about the experimental protocol and schedule the first experimental session.

**Fig. 1:**
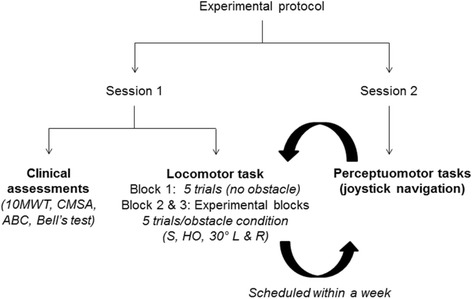
Flowchart describing the experimental protocol. 10MWT: 10-min walk test; CMSA: Chedoke McMaster Stroke Assessment; ABC: Activities-Specific Balance Confidence scale; S: stationary, HO: head-on, L:left; R:right. Note that results from the perceptuomotor task are not included in this study

Twelve participants with chronic stroke (40–70 years of age) and 12 age-matched healthy controls without any self-reported premorbid conditions that interfered with walking participated in this study, in the period between July 2012 and August 2014. Stroke participants were included if they had a first incidence of supratentorial stroke in the middle cerebral artery territory with an onset of more than 6 months, walking 30 m independently with or without use of a cane, scoring > 27 on the Mini-Mental State Examination [9] obtained from the medical charts, and staging 3/7 or higher on the leg component of the Chedoke McMaster Stroke Assessment (CMSA) [10]). Excluded were those with visuospatial neglect (as screened by Bell’s test [11]) and visual field deficits (reported in medical charts). After screening for exclusion criteria, each participant signed an informed consent form as approved by the research ethics board. Clinical assessment performed in the first session (Fig. 1) included comfortable gait speed using the 10 m walk test [12], motor ability of the lower limbs measured with the help of CMSA (leg and foot impairment stages) [10], cognitive ability using the Montreal Cognitive Assessment (MoCA; [13] and balance confidence measured using the Activities Balance Confidence Scale (ABC; [14, 15]).

### Virtual environment (VE)

The VE, viewed through a helmet-mounted display (HMD; Kaiser Electro- Optics, Carlsbad, CA, USA; FOV: 50° diagonal; screen resolution: 1280 × 1024 pixels), consisted of a room (11 × 8 m) with three red cylindrical obstacles (arranged around an arc of radius 4 m) and a central blue target located at the far end [8]. Participants were asked to walk in the virtual room at a comfortable pace towards the target while avoiding collisions with the obstacles. After advancing 0.5 m, one of the obstacles moved randomly at a speed of 0.75 m/s towards a pre-determined point of intersection (PoI) from head-on, 30° left or right. The PoI was defined as the theoretical point of intersection of the obstacle and participant trajectories (if no avoidance strategy was undertaken and participants walked in a straight line towards the target), and was located at 4 m from the obstacles and the participants’ initial position. In the stationary obstacle condition, the red cylinder stayed still at the PoI. Each obstacle condition (stationary, moving head-on or diagonally left/right) was randomly repeated for 5 trials each over two blocks, with the inclusion of 5 control walking trials with no obstacles presented at the beginning and the end. (Fig. 1).

Reflective markers were placed on 41 pre-determined anatomical locations of the body (Vicon Plug-In-Gait model [16, 17]) and recorded at 120 Hz with a 12-camera motion capture system (512 Workstation, Vicon Motion Systems Ltd. UK). The real-time, 3D positions of three head markers, placed on the HMD, were also used to synchronize motion in the virtual scene with physical movement of the participants in the laboratory space.

### Data processing

After reconstruction of marker positions in 3D, data were filtered using a fourth order, dual - pass Butterworth filter at 6 Hz, and CoM and joint angles (specifically segmental rotations), were extracted from the processed trial data.

**Fig. 2:**
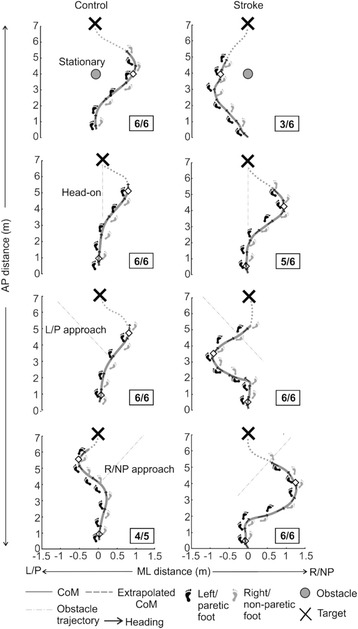
Representative trial demonstrating body center of mass (CoM) displacement in the stationary, head-on, left/paretic and right/non-paretic approach in a control (left panels) and stroke (L paretic) participant (right panel). Also plotted are the foot trajectories (L/P foot: black; R/NP foot: grey) and heading direction (black arrows). The foot placements represent the stance phase for each foot while the heading direction is plotted every 1 s. Unfilled diamonds diamonds signify spatial position when obstacle motion is initiated. Filled white diamonds signify spatial position at which antero-posterior positions of the participant and obstacle are the same (crossing point). The number of trials where circumvention to the side indicated in the figure is mentioned in boxes next to individual figure panels

Figure 2 illustrates CoM displacements in the anteroposterior (AP) and mediolateral (ML) direction in representative trials from one post-stroke (left paretic) and one healthy control for all obstacle conditions. As the avoidance strategy commenced after initiation of obstacle motion and ended when both obstacle and the participant were at a similar AP position (i.e., at obstacle crossing), further analysis of data was confined between these time periods. Feet marker data were used to generate step width at mid-stance phase at each step for each foot using customized Matlab scripts (Mathworks Inc., MA, USA). For straight walking, step width for each lower limb was simply computed as the distance between the two heel markers in the mid-stance phase. For curved walking as seen in the present study, a point equidistant from the toe and heel marker was identified for each foot as the mid-point. Further, for each foot, a stride vector was identified as the vector representing change in heel marker position between subsequent gait cycles. Step width at mid-stance for each foot was then calculated as the perpendicular distance from the mid-point of the contralateral foot to the stride vector. For instance, left foot width was the perpendicular distance from the mid-point of the right foot to the left stride vector.

### Outcomes

**Fig. 3:**
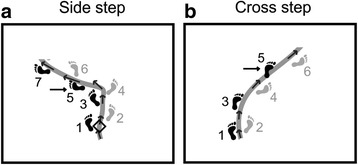
Examples of stepping strategies (**a** side step strategy, **b** cross step strategy). Arrows point towards the foot that initiated the strategy
- Segmental rotation: maximum horizontal rotation (yaw) amplitudes of the head, thorax, pelvis and left and right feet, as well as thorax roll amplitude.
- Onset of segment reorientation: identified as the point in the first half of a trial (beginning from the initiation of walking for stationary obstacle conditions and from the onset of obstacle motion for moving obstacle conditions) which marked the onset of change > 2SD above the average change in corresponding segment angles recorded in the no-obstacle trials.

The main outcome measures included:

- Stepping strategy: The foot that initiated the stepping strategy was identified as the one that led a change in step width (leading foot), above or below one SD of the step width observed in the no-obstacle trials. A side step strategy was identified when the leading foot was ipsilateral to the side of circumvention and demonstrated an increase in step width to change the steering direction (Fig. 3a). A cross step strategy was identified when the leading foot was contralateral to the side of circumvention and demonstrated a reduction in step width to change the steering direction (Fig. 3b).

### Statistical analyses

Data reported in this study includes only successful non-collision trials. Stationary vs. head-on and diagonal approaches were analyzed separately. Dependent variables were examined for normality using tests of skewness, kurtosis, Shapiro-Wilk test and Q-Q plots. Data were considered appropriate for analysis by parametric tests if the skewness value was between ±2 [18], if the Shapiro-Wilk test was non-significant and if the spread of data on the Q-Q plot did not deviate far from the expected normal slope. All data used for analysis in this study met these parameters. Between group differences (stroke vs. healthy) with respect to participants’ age and clinical assessments (gait speed, MoCA and ABC scores) were analyzed using unpaired t-tests. The proportion of side step vs. cross step strategy for each group and obstacle condition was analyzed separately using the paired *t*-test. Separate two-way (2 × 2) repeated measures mixed model analyses were used to determine the impact of group (stroke vs. control) and obstacle approach (stationary vs. head-on) on head, thorax, pelvis, L/P foot, R/NP foot yaw and thorax roll amplitudes, respectively. Similarly, separate two-way repeated measures mixed model analyses were used to determine the effects of group and obstacle approach (R/NP vs. L/P) on joint amplitudes for the above mentioned segments except thorax roll (as thorax roll was not significantly different for both approaches as compared to the no-obstacle trials). Two-way repeated measures mixed model analyses were used to determine impact of group and joint segment on reorientation onsets for stationary and head-on approaches separately (as onset of segment reorientation was identified differently for these conditions). A three-way 2 × 2 × 5 repeated measures mixed model analysis was performed to detect differences in reorientation onsets among groups (stroke vs. control), obstacle approach (L/P vs. R/NP) and joint segments (head, thorax, pelvis, L/P foot and R/NP foot rotations) for diagonal obstacle approaches. Bonferroni adjustments were used for all repeated measures analyses, with significance set at *p* < 0.05. All statistical analyses were performed using the IBM SPSS Statistics 20 software (IBM Corporation, NY, USA).

## Results

### Participants

On an average, the stroke group was slightly older (healthy: 52.5 ± 8.3 years; stroke: 56.0 ± 7.0 years, *p* = 0.29) and consisted of more males than females as compared to healthy participants (healthy: 8 males/4 females; stroke: 10 males/2 females, *p* = 0.37), however this difference was not statistically significant. Stroke participants walked significantly slower as compared to healthy controls (control: 1.49 ± 0.21 m/s; stroke: 0.86 ± 0.38 m/s, *p* < 0.05) and also had significantly lower scores on the MoCA (control: 28.25 ± 1.29; stroke: 24.08 ± 2.79, *p* < 0.05) and on the ABC (control: 94.47 ± 5.95%; 69.34 ± 15.57%, *p* < 0.05) as compared with healthy controls (Table 1). Also, half of the post-stroke participants used a walking aid (cane) habitually and while performing the experiment.

**Table 1:** Stroke participant characteristics

	Age (yrs)	Time since stroke onset (yrs)	Side of lesion (R/L)	Gait speed (m/s)	Gait Speed in VE (m/s)	CMSA (/7)		ABC (%)	Cane use (+/−)
						Leg	Foot		
*S1*	46	2.5	R	0.68	0.23	5	4	60.63	+
*S2*	54	4	R	0.31	0.26	3	2	71.25	+
*S3*	59	3	R	0.42	0.55	5	3	86.88	+
*S4*	60	2	L	0.36	0.2	3	3	50.94	+
*S5*	51	6	L	0.9	0.66	4	2	48.13	+
*S6*	54	2.5	R	1.27	0.67	5	4	83.13	-
*S7*	68	1.5	L	0.73	0.46	5	5	67.50	-
*S8*	48	2	L	1.15	0.63	4	4	71.25	+
*S9*	52	2.75	L	1.36	1.04	7	6	90.31	-
*S10*	62	1	R	0.7	0.77	6	5	64.06	-
*S11*	66	7	R	1.3	0.93	6	5	89.38	-
*S12*	51	5	L	1.09	0.71	5	4	48.75	+
*Stroke* *(Mean (SD))*	56.0 (7.0)	3.3 (1.9)		0.86 (0.38)*	0.59 (0.27)			69.34 (15.57)*	6/12
*Controls* *(Mean (SD))*	52.5 (8.3)	--	--	0.49 (0.21)	0.98 (0.14)	--	--	94.47 (5.95)	0/12

Included at the bottom are the mean demographic information and scores of the stroke and control participants for comparison, *p < 0.05, yrs: years, *VE* Virtual environment, *CMSA* Chedoke – McMaster Stroke Assessment, *ABC* Activities Specific Balance Confidence scale, *R* Right, *L* Left

### Stationary vs. mobile (head-on) approach

#### Stepping strategy

In healthy controls, the side step strategy was used in a slightly larger proportion of trials in both stationary (57.92 ± 18.76%) and moving (65.69 ± 25.27%) obstacle conditions as compared to the cross step strategy. This difference was however not statistically significant. Similarly, in comparison with the cross step strategy, the side step strategy was preferred by post-stroke participants for stationary (67.78 ± 30.69%) and to a significantly larger extent for moving (75.56 ± 30.42%, *p* < 0.05, Cohen’s d = 0.84) obstacle conditions.

#### Segmental coordination

**Fig. 4:**
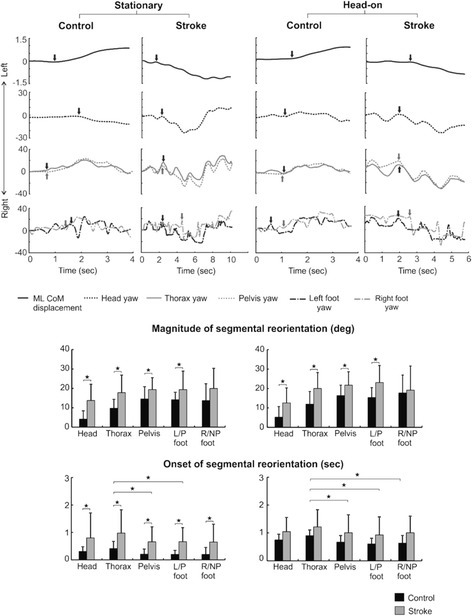
Segmental horizontal rotations from one representative trial each from a control and post-stroke (right paretic) participant for stationary and head-on obstacle conditions. Vertical arrows indicate onset of segmental reorientation. Bar graphs represent average yaw amplitudes and onsets of segment reorientation in control and post-stroke participants. Error bars represent SD. * *p* < 0.05

Maximum amplitudes for all joint segments during circumvention were significantly greater in stroke as compared with healthy participants (head yaw: df = 22, F = 10.646, *p* = 0.004, ω_p_^2^ = 0.287; thorax yaw: df = 22, F = 8.779, *p* = 0.007, ω_p_^2^ = 0.245; thorax roll: df = 22, F = 26.097, *p* = 0.001, ω_p_^2^ = 0.511; pelvis yaw: df = 22, F = 5.442, *p* = 0.029, ω_p_^2^ = 0.155; left/P foot yaw: df = 22, F = 6.008, *p* = 0.023, ω_p_^2^ = 0.173; Fig. 4). However, segmental amplitudes were not significantly different between stationary and head-on obstacle conditions.

For the stationary obstacle, stroke participants initiated segmental reorientation later than healthy participants (df = 20, F = 5.949, *p* = 0.024, ω_p_^2^ = 0.184), and thorax rotation was initiated significantly later than both pelvis and L/P foot rotation in both groups (df = 20, F = 18.984, *p* < 0.001, ω_p_^2^ = 0.776; Fig. 4). For the head-on approach, although segment reorientation was initiated later in the stroke group than healthy participants, the difference was not statistically significant. However, thorax yaw was initiated significantly later than pelvis and feet rotation (df = 22, F = 37.416, *p* < 0.001, ω_p_^2^ = 0.867). In both control and stroke groups, a similar sequence of segmental reorientation was found where initiation of avoidance strategy was led by one foot, followed closely with the pelvis and the other foot. Thorax roll and thorax yaw followed the lower body while head yaw lagged or preceded thorax yaw.

### Diagonal obstacle approach

The preferred side of circumvention when obstacles approached diagonally differed between control and post-stroke participants. While control participants circumvented to the opposite side of obstacle approach in more than 60% trials, 7/12 stroke participants circumvented to the same side as obstacle approach (V_same_; S1-S7; Table 2) and 5/12 participants circumvented to the opposite side of obstacle approach (V_opp_; S8-S12; Table 1). In comparison with the V_opp_ group, stroke participants in the V_same_ group demonstrated lower gait speeds, increased restrictions in motor ability as indicated by lower stages of the CMSA, impaired balance indicated by habitual cane use and reduced balance confidence indicated by lower scores on the ABC (Tables 1 and 2). Coordination strategies were different for these sub-groups, as described below.

**Table 2:** Clinical assessment scores of participants (Mean ± SD)

	Age (years)	Time since stroke onset (years)	Gait speed (m/s)	Gait speed in VE (m/s)	CMSA (/7)		ABC (%)
					Leg	Foot	
Controls	52.50 ± 8.3	--	1.49 ± 0.21	0.98 ± 0.14	--	--	94.47 ± 5.95
Stroke S1-S7 (V_same_)	56.00 ± 7.09	3.07 ± 1.51	0.67 ± 0.34	0.43 ± 0.20	4.29 ± 0.95	3.29 ± 1.11	66.92 ± 14.88
Stroke S8-S12 (V_opp_)	55.80 ± 7.76	3.55 ± 2.43	1.12 ± 0.26	0.82 ± 0.17	5.7 ± 1.14	4.8 ± 0.84	72.75 ± 17.60

### Stepping strategy

Both side step and cross step strategies were equally favored for both left (side-step: 50.58 ± 32.94%, cross step: 37.76 ± 33.77%) and right (side-step: 44.03 ± 37.03%, cross step: 44.86 ± 35.78%) obstacle approaches in control participants. No veering and stepping changes were found in a small proportion of trials as well (~11.0 ± 30.0% for both approaches). A significantly large proportion of side step strategy was seen among stroke participants for the obstacle approaching from the paretic side (side step: 67.08 ± 35.49%, cross step: 32.92 ± 35.49%, *p* < 0.05, Cohen’s d = 0.99). The difference between stepping strategies was, however, not significant for the non-paretic obstacle approach. Among stroke participants, V_same_ sub-group favored the side-step strategy for both P (75%) and NP (80%) obstacle approaches, while the V_opp_ sub-group showed a larger proportion of side step strategy (56%) for the P-sided obstacle approach, and the cross step strategy (62%) for the NP-sided obstacle approach.

### Segmental reorientation

**Fig. 5:**
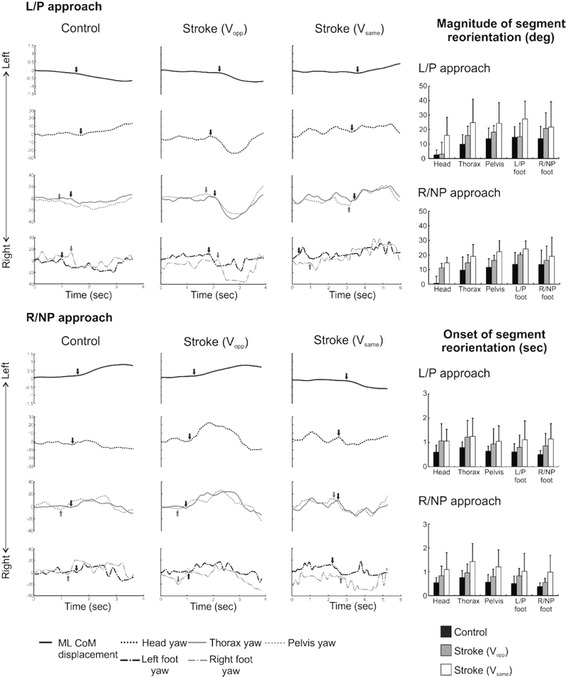
Segmental horizontal rotations from one representative trial each from a control and two post-stroke participants (both left paretic) - one each from the V_opp_ and V_same_ sub-groups for left/paretic and right/non-paretic obstacle approaches. Vertical arrows indicate onset of segmental reorientation. Bar graphs represent average yaw amplitudes and onsets of segment reorientations in control and post-stroke (V_opp_ and V_same_) participants. Error bars represent SD

For both paretic and non-paretic obstacle approaches, stroke participants demonstrated significantly larger horizontal rotation amplitudes for most joint segments as compared to controls (head: df = 22, F = 8.251, *p* = 0.009, ω_p_^2^ = 0.240, thorax: df = 22, F = 10.632, *p* = 0.004, ω_p_^2^ = 0.295, pelvis: df = 22, F = 9.020, *p* = 0.007, ω_p_^2^ = 0.259 and L/P foot: df = 22, F = 11.003, *p* = 0.003, ω_p_^2^ = 0.303; Fig. 5). Stroke participants also initiated segmental orientation significantly later than control participants (df = 21, F = 8.520, *p* = 0.008, ω_p_^2^ = 0.246). Further, in both stroke and control groups, thorax yaw was initiated significantly later than pelvis and feet yaw (df = 21, F = 24.781, *p* = 0.000, ω_p_^2^ = 0.785; Fig. 5), indicating a caudal-rostral sequence of reorientation.

Amongst stroke participants, for both paretic and non-paretic sided obstacle approach, the V_same_ sub-group employed larger segmental rotations and initiated segmental rotations later as compared to the V_opp_ group (Fig. 5). Further, it is noteworthy that although the control and the V_opp_ groups opted to circumvent to the opposite side of the obstacle approach, the yaw amplitudes seen in V_opp_ stroke participants were greater than the controls.

In summary, in stroke participants, the preferred stepping strategies differed depending upon obstacle approach direction and the preferred side of circumvention. Also, for all obstacle contexts, post-stroke participants demonstrated greater amplitudes and later onset of segmental rotation as compared with healthy controls.

## Discussion

By contrasting obstacle circumvention strategies between post-stroke and healthy individuals, we show that post-stroke individuals use altered segmental coordination and step strategies for obstacle circumvention as compared to healthy individuals and that these strategies may be influenced by obstacle contexts as well as functional abilities of post-stroke individuals.

### Stationary and moving (head-on approach) obstacle

The side step strategy was preferred to initiate obstacle circumvention for both stationary and moving obstacle, but more so for the moving obstacle conditions by post-stroke individuals. This strategy involves an increase in step width (and base of support) and may have been employed to enhance dynamic stability during a destabilizing task such as execution of a transient path deviation. Individuals with stroke demonstrate decreased dynamic stability during locomotion [19] and are known to increase step width to counter perturbations while walking [20]. A similar strategy may have been utilized for obstacle circumvention.

Stroke participants executed significantly larger horizontal segmental rotations to circumvent stationary obstacles. This may have been used to maintain larger clearances when crossing the obstacle [21]. The segmental rotations, although greater in stroke participants than controls, were not significantly different between the two groups, when encountered with a head-on moving obstacle. Also, the clearance at obstacle crossing was smaller in the stroke group [21], indicating although an attempt to employ greater rotations possibly to increase clearance was made; it did not lead to desired larger clearance at crossing.

Segmental rotations were initiated later in post-stroke individuals than controls. This could have resulted from difficulty in executing rapid gait adjustments in response to presence of an external stimulus such as a moving obstacle [22–25].

### Diagonal obstacle approaches

Two distinct sub-groups amongst stroke participants were revealed when obstacles approached diagonally - V_opp_ (S8-S12; Table 1) and V_same_ (S1-S7). The V_same_ group thus had greater functional limitations as compared to the V_opp_ group as indicated by increased limitations demonstrated in clinical assessments. Concurrent with these differences in functional abilities, the V_same_ and V_opp_ sub-groups were found to adopt different stepping and coordination strategies.

As compared to control participants who used the side and cross step strategies to similar extents, the V_opp_ group preferred the side step strategy for P-sided obstacle approach (to veer to the NP side) and the cross step strategy for NP-sided approach (to veer to the P side). Both strategies were thus led by the NP foot. Since the V_opp_ group veered towards the opposite side of obstacle approach (thus passing in front of the obstacle), a rapid avoidance response was necessary to avoid a collision. Individuals with stroke prefer the non-paretic foot to execute time-bound rapid stepping responses [26]. A similar strategy was noted in this study where relatively quicker gait adaptations were required.

In contrast, the V_same_ group preferred the side step strategy irrespective of the obstacle approach direction. Since the V_same_ group had greater restrictions in motor ability and compromised balance capabilities (indicated by cane use); a side step response may have been used to enhance stability while executing a potentially destabilizing transient change in walking direction. Interestingly, cane use did not seem to have an effect on the choice of stepping strategy in the V_same_ group. For instance, if the physical presence of a cane had an influence on the choice of stepping strategy, individuals in the V_same_ group (who used the cane on the non-paretic side) would have chosen the cross step strategy initiated with the paretic lower limb when circumventing to the non-paretic side. However, this group consistently used the side step strategy irrespective of the presence of a cane, suggesting that cane use may not have had a direct impact on the choice of stepping strategy. Nevertheless, it should be noted that cane use and choice of the side step strategy may be indicators of impairments in balance especially during locomotor adaptations such as those required during obstacle circumvention.

Segmental rotations in the V_same_ group were larger than both controls and the V_opp_ group. Considering that clearance was also greater in this sub-group [21], the larger yaw amplitudes may have been employed to execute larger clearances. The trend of increasing delays in segmental reorientation from the control to the V_opp_ and V_same_ group is indicative of increasing difficulties with postural reorientations during locomotion [27].

Interestingly, the V_opp_ group, despite executing a similar circumvention strategy as controls, demonstrated larger yaw amplitudes and later onsets of segmental reorientation. This suggests that high functioning post-stroke individuals, who employ similar adaptation strategies as healthy individuals, may still show deficient coordination strategies. Similarly, altered coordination strategies were also found in well-recovered post-stroke individuals when voluntarily executing intended turns to change walking direction [27, 28].

### Segmental reorientation sequence

Coordination strategies used while executing a transient change in walking direction while circumventing obstacles are different from those used while changing walking direction to steer towards a new goal [3]. This was also seen in the present study where the segmental reorientation sequence was led by the feet and followed a caudal-rostral sequence in both control and stroke participants. This greater contribution of the feet to affect a directional change is also in agreement with a previous study [3] conducted with healthy adults. Similar reorientation sequences in controls and post-stroke participants suggest that despite larger rotations and later onsets of segment reorientations, the underlying reorientation sequence may have been conserved in the post-stroke population [24].

In summary, when executing avoidance strategies in the presence of obstacles, individuals with stroke use larger yaw amplitudes to maintain larger clearances and side stepping that enhance stability when performing a destabilizing task such as changing walking direction.

### Clinical implications

Independent community ambulation is an important goal for stroke survivors. Assessment and training of complex locomotor tasks encountered in the community are imperative in facilitating this important objective. Both stationary and moving obstacles are encountered frequently in the community, yet obstacle circumvention is rarely assessed or trained in rehabilitation settings [28]. The present study provides evidence that individuals with chronic stroke but without visuospatial perception and cognitive impairments are most likely to choose strategies that ensure success (collision-free avoidance). These strategies emerge from an interaction between personal (functional limitations) and environmental constraints (obstacle conditions). Assessment and intervention plans that target obstacle circumvention should therefore take both personal and environmental factors into consideration to customize treatment plans for each individual.

## Conclusion

Altered postural coordination strategies were used by post-stroke individuals during obstacle circumvention. Obstacle circumvention strategies were thus influenced by obstacle characteristics (suggested by difference in clearances dependent upon obstacle conditions) as well as an individual’s functional abilities, evidenced by difference in clearance, stepping strategies as well as amount and onset of segmental orientation amongst individuals with stroke with differing functional abilities.

## Abbreviations

ABC: Activities-specific Balance Confidence scale
AP: Aneroposterior
CMSA: Chedoke-McMaster Stroke Assessment
CoM: Center of mass
HMD: Helmet-mounted display
L: Left
ML: Mediolateral
MMSE: Mini-Mental Scale Examination
NP: Non-paretic
P: Paretic
PoI: Point of intersection
R: Right
SD: Standard deviation
VE: Virtual environment
